# High-Resolution Crystal Structure of Chloroplastic Ribose-5-Phosphate Isomerase from *Chlamydomonas reinhardtii*—An Enzyme Involved in the Photosynthetic Calvin-Benson Cycle

**DOI:** 10.3390/ijms21207787

**Published:** 2020-10-21

**Authors:** Théo Le Moigne, Pierre Crozet, Stéphane D. Lemaire, Julien Henri

**Affiliations:** 1 Laboratoire de Biologie Moléculaire et Cellulaire des Eucaryotes, UMR8226, Institut de Biologie Physico-Chimique, Sorbonne Université, CNRS, 75005 Paris, France; lemoigne@ibpc.fr (T.L.M.); pierre.crozet@sorbonne-universite.fr (P.C.); lemaire@ibpc.fr (S.D.L.); 2 Faculty of Sciences, Doctoral School of Plant Sciences, Université Paris-Saclay, 91190 Saint-Aubin, France; 3 Polytech Sorbonne, Sorbonne Université, 4 place Jussieu, 75005 Paris, France; 4 Laboratoire de Biologie Computationnelle et Quantitative, UMR7238, Institut de Biologie Paris-Seine, Sorbonne Université, CNRS, 4 place Jussieu, 75005 Paris, France

**Keywords:** photosynthesis, chloroplast, Calvin–Benson cycle, protein structure, X-ray crystallography, ribose isomerase, post-translational modification, redox, oligomerization, protein complexes

## Abstract

The Calvin–Benson cycle is the key metabolic pathway of photosynthesis responsible for carbon fixation and relies on eleven conserved enzymes. Ribose-5-phosphate isomerase (RPI) isomerizes ribose-5-phosphate into ribulose-5-phosphate and contributes to the regeneration of the Rubisco substrate. Plant RPI is the target of diverse post-translational modifications including phosphorylation and thiol-based modifications to presumably adjust its activity to the photosynthetic electron flow. Here, we describe the first experimental structure of a photosynthetic RPI at 1.4 Å resolution. Our structure confirms the composition of the catalytic pocket of the enzyme. We describe the homo-dimeric state of the protein that we observed in the crystal and in solution. We also map the positions of previously reported post-translational modifications and propose mechanisms by which they may impact the catalytic parameters. The structural data will inform the biochemical modeling of photosynthesis.

## 1. Introduction

Photosynthesis is the biological process allowing the conversion of light energy into chemical energy through fixation of atmospheric carbon [1]. Fixation of carbon dioxide occurs through the Calvin–Benson cycle (CBC) by carboxylation of the acceptor pentose ribulose-1,5-bisphosphate (RubP) by Rubisco [2]. RubP is generated by phosphorylation of ribulose-5-phosphate (Ru5P) by phosphoribulokinase (PRK). Prior to this phosphorylation step, ribose-5-phosphate (R5P) carbonyl group is transferred from carbon 1 to carbon 2. This reaction is catalyzed by the metabolic enzyme ribose-5-phosphate isomerase (RPI) [3]. Two forms of RPI have been classified: type A (RPI_A_) and type B (RPI_B_). RPI_A_ is ubiquitous in all species while RPI_B_ is only present in some bacteria and protozoans [4]. Beside its critical role in the CBC, RPI_A_ is implicated in the non-oxidative branch of the pentose phosphate pathway (PPP) [5]. RPI_A_ function in the PPP makes it an attractive target for drug development in the treatment of trypanosomatid-caused diseases [4]. RPI_A_ structures and catalytic mechanism (EC 5.3.1.6) have been described in several organisms, but the structure of photosynthetic chloroplast localized RPI_A_ has never been reported to date. Analysis of RPI enzymatic kinetics has been principally performed on RPI_B_ in several species, and research on RPI_A_ enzymology is comparatively scarcer. The catalytic mechanism of RPI_A_ has nonetheless been established in several species and summarized in a recent review [4].

*Chlamydomonas reinhardtii* is a model unicellular photosynthetic green alga [6]. Its nuclear genome encodes two RPI at loci Cre03.g187450.t1.2 and Cre07.g314600.t1.2 [7], the protein products of which are respectively named RPI1 and RPI2. Both proteins are predicted to be addressed to the chloroplast according to the Predalgo tool [8] and in agreement with the chloroplast localization of the CBC and PPP in *C. reinhardtii* [9,10]. Among RPI1 and RPI2, RPI1 was selected as the most probable contributor to the CBC because its transcripts are 16 to 199 times more abundant than that of RPI2 in *C. reinhardtii* [11] and because accumulation of RPI1 transcript varies according to a night/day cycle, mirroring luminous flux.

In *C. reinhardtii*, the estimated CrRPI1 protein quantity is 0.34 amol per cell and 2.5 µmol.L^−1^ in the chloroplast [12]. Considering the cellular concentration of metabolites [12,13], the approximate molar ratio of substrate or product to CrRPI1 is respectively estimated at 80 and 50. In order to adapt to the variations of metabolites concentration in the chloroplast stroma, CBC enzymatic activities are generally regulated according to the environmental light [14,15]. These regulations occur through several signaling pathways, e.g., dithiol-disulfide exchanges, S-thiolations, S-glutathionylations, and S-nitrosylations [15,16,17]. The stroma magnesium concentration is also known to influence the catalytic activity of CBC enzymes [18,19]. Post-translational redox modifications of RPI were detected from *C. reinhardtii* proteomic studies [20]. Phosphorylated peptides of CBC enzymes were also identified from *C. reinhardtii* extracts [21], suggesting a catalytic post-translational regulation by reversible phosphorylation.

Here, we present the first experimental structure of a chloroplastic RPI. We describe the structural characteristics that classifies CrRPI1 in the RPI_A_ family. We map the positions of previously reported post-translational modifications and propose mechanisms by which they may impact the catalytic parameters.

## 2. Results

### 2.1. CrRPI1 Structural Folds

**Figure 1:**
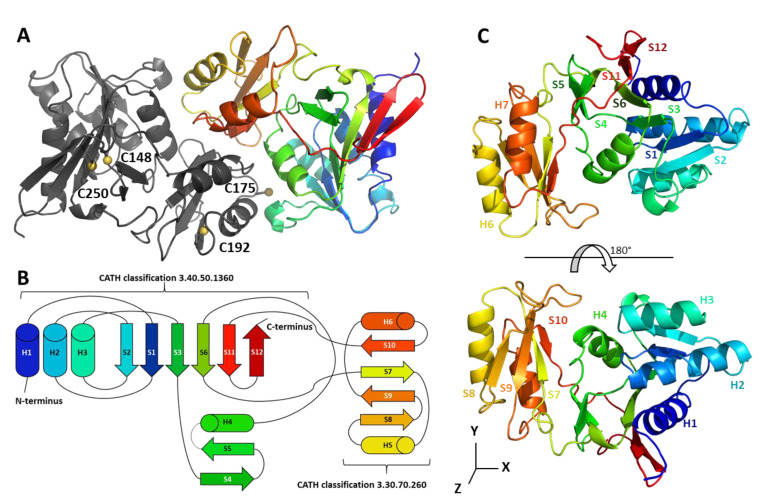
Crystal structure of CrRPI1. (**A**) Cartoon representation of the CrRPI1 crystallographic dimer colored in grey for the first subunit and from blue (N-terminus) to red (C-terminus) for the second subunit. (**B**) Two-dimensional representation of secondary structures of CrRPI1 colored as the second subunit in (**A**). (**C**) Cartoon representation of a CrRPI1 monomer. Two views of the protein are represented as in (**A**) and rotated by 180° on the *x*-axis. Secondary structures are annotated as in (**A**).

We solved the crystal structure of CrRPI1 in the P2_1_ space group at 1.40 Å resolution (Table 1). Model building of the two subunits of the asymmetric unit achieved the placement of 235 amino acid residues in the first subunit and 237 in the second (Figure 1A). The residues of each subunit were numbered according to uniProtKB entry A8IRQ1. The high resolution of the dataset further allowed us to position eight sulfates, two sodiums, and 511 water molecules in the crystal asymmetric unit.

Each monomer is composed of six alpha helices and twelve beta strands distributed into two domains. Domain 1 spans residues 29 to 160 and 246 to 264, folds according to the Rossmann topology, and is part of an unnamed structural superfamily (CATH classification 3.40.50.1360). Domain 2 is inserted in between the two parts of domain 1, spans residues 161 to 245, and is classified as an ACT domain (CATH classification 3.30.70.260) (Figure 1B).

Our model aligns with 62 entries of the Protein data bank with an average RMSD lower than 1.8 Å. The closest similarity is with the Ribose 5-phosphate isomerase from *Plasmodium falciparum* (PDB 2F8M, RMSD = 0.852 Å over 229 aligned Cα). CrRPI1 secondary structure content is distributed as follows: α helix h1 (residues 33–45), β strand s1 (52–56), h2 (60–75), s2 (81–85), h3 (88–97), s3 (112–115), s4 (118–121), s5 (124–127), h4 (135–143), s6 (146–152), s7 (167–171), h5 (176–185), s8 (193–196), s9 (211–216), h6 (225–233), s10 (238–240), s11 (249–254) and s12 (257–262) (Figure 1C). In addition, residues 104, 105, 106 and residues 152, 153, 154 form two 3_10_ helices. Residues 22 to 28 and 265 to 269 at amino- and carboxy-termini were not modelled because of a lack of interpretable electron density. The secondary structure content and organization is the same as a canonical RPI previously reported (PfRPI, PDB 2F8M, [22]) except for the presence of CrRPI1 3_10_ helix formed by residues 104–106 that is absent from PfRPI.

Domain 1 is composed of helices 1, 2, 3, and 4, with strands 4 and 5 forming a small antiparallel sheet and strands 1, 2, 3, 6, and 12 that arrange in a parallel sheet completed by strand 11 in antiparallel orientation. These secondary structures are organized in a three-layer sandwich composed of helices 1, 2, 3 for the first layer, the parallel sheet in the second layer and by strand 4, 5, helix 4 and the two 3_10_ helices for the third layer. Domain 2 is relatively smaller with helices 5 and 6 forming the first stratum of a two-layer sandwich and strands 7, 8, 9, and 10 organized as an antiparallel sheet forming the second layer. The first domain contains the catalytic pocket, while the second was proposed to play a role in the oligomerization of RPI_A_ [4].

### 2.2. CrRPI1 is a Homodimer

**Figure 2:**
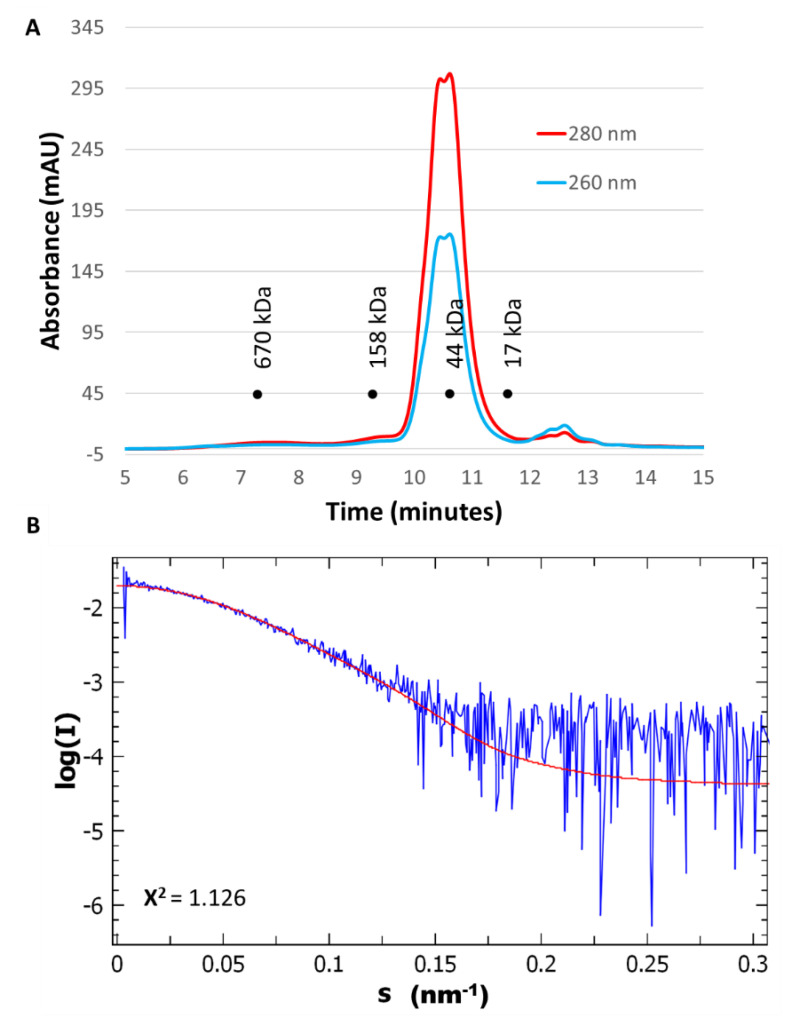
Size-exclusion chromatography coupled to small angle X-rays scattering (SEC-SAXS) analysis of CrRPI1. (**A**) High performance liquid chromatography (HPLC) chromatogram of CrRPI1 with absorption at 280 nm in red and at 260 nm in blue. (**B**) SAXS scattering curve log(I) = f(s) in blue was fitted to the crystallographic dimeric model of CrRPI1 in red.

**Figure 3:**
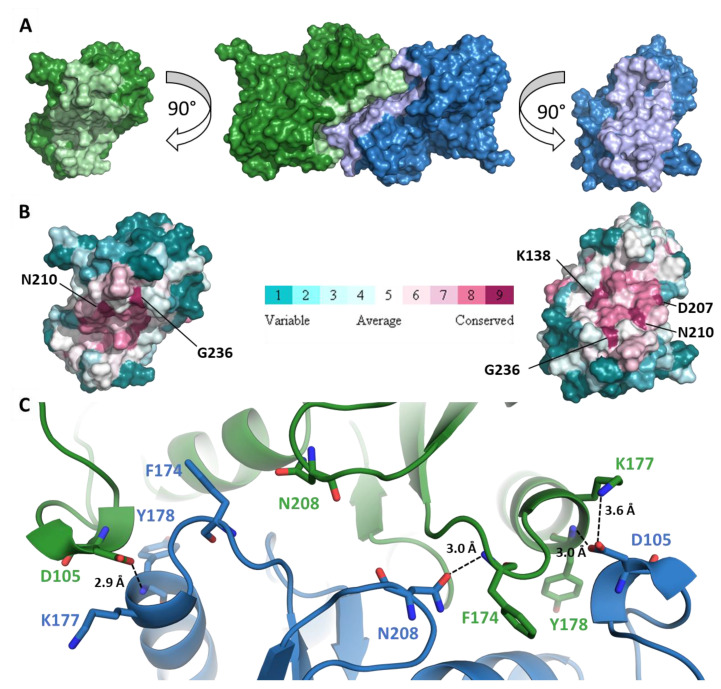
CrRPI1 homodimerization. (**A**) From left to right, Connolly solvent exclusion surface representation of subunit A, dimer and subunit B of CrRPI1. Subunit A is colored in green, and subunit B in blue. Residues composing the interface between the two subunits are colored in light green and light blue for subunits A and B respectively. (**B**) Surface residues conservation of subunits A and B as calculated with CONSURF colored from teal (least conserved) to purple (most conserved). Most conserved residues of the interface are annotated. (**C**) Cartoon representation of the dimer interface. Residues in bonding distances are represented in stick and annotated. Polar contacts are traced with black dashed lines with interatomic distances indicated in Ångström (Å).

RPI_A_ has been described as a functional dimer in solution [3,23,24] in bacteria and protozoa or as a functional tetramer in species from archaea and fungi taxa [4]. However, nothing was described for *Viridiplantae* enzymes. In our study, CrRPI1 elutes with the apparent molecular weight of a homodimer in solution over two different gel filtration matrices (Figure 2A). Small angle X-rays scattering (SAXS) curves obtained after size-exclusion chromatography of the purified protein (SEC-SAXS, Figure 2B) compute a molecular weight between 49.25 kDa and 56.2 kDa, fitting with two monomers of 27.55 kDa each. The homodimeric state is also observed in the crystal asymmetric unit (Figure 1A) with α helices h4, h5 and h6 and β strands s10 contributing to the interface. CrRPI1 dimer was aligned with *Plasmodium falciparum* RPI dimer (PDB entry 2F8M) with an RMSD = 2.115 Å over 438 equivalent atoms. Both dimers have the same quaternary organization, with corresponding subunits positioned in virtually identical positions. The only significant difference lies in the 3_10_ helix formed by residues 104–106 of CrRPI1 that is structured as a loop in PfRPI. The interface between two subunits of CrRPI1 is calculated from the arrangement of the two subunits forming the asymmetric unit (Figure 3A) by the PDBe-PISA server v1.52 [25]. The subunit A-to-subunit B interface is 1165 Å^2^ wide and has a computed Δ^i^G = −19.3 kcal/mol (*p*-value = 0.027). In comparison, PfRPI dimer interface is more extended with a surface of 1340.8 Å^2^ and an equivalent computed Δ^i^G = −19.4 kcal/mol (*p*-value = 0.048). CrRPI1 interface is composed by the 33 following residues: P85, T103, L104, D105, P108, K109, L135, R136, K138, M139, V140, M142, A143, V172, Q173, F174, C175, H176, K177, Y178, T179, R82, D207, N208, S209, N210, L232, G233, D235, G236, V237, V238, D239. It is stabilized by three hydrogen bonds between residues Y178_A_ (α helix h5) and D105_B_ (3_10_ helix 104–106), F174_A_ and N208_B_, D105_A_ and Y178_B_, and one salt bridge between K177_A_ and D105_B_ (Figure 3C). PfRPI possesses similar pairs Y145_A_–E74_B_, N177_A_–F143_B_, and E74_A_–K146_B_ (main chain carbonyl) in bonding distance, but no salt bridge and additional hydrogen bonds that are absent in CrRPI1 homodimer. While CrRPI1 dimerization interface residues are strictly conserved in the algae taxon, they are relatively poorly conserved throughout evolution (Figure 3B and Figure S1) [26].

### 2.3. RPI1 Catalytic Site

**Figure 4:**
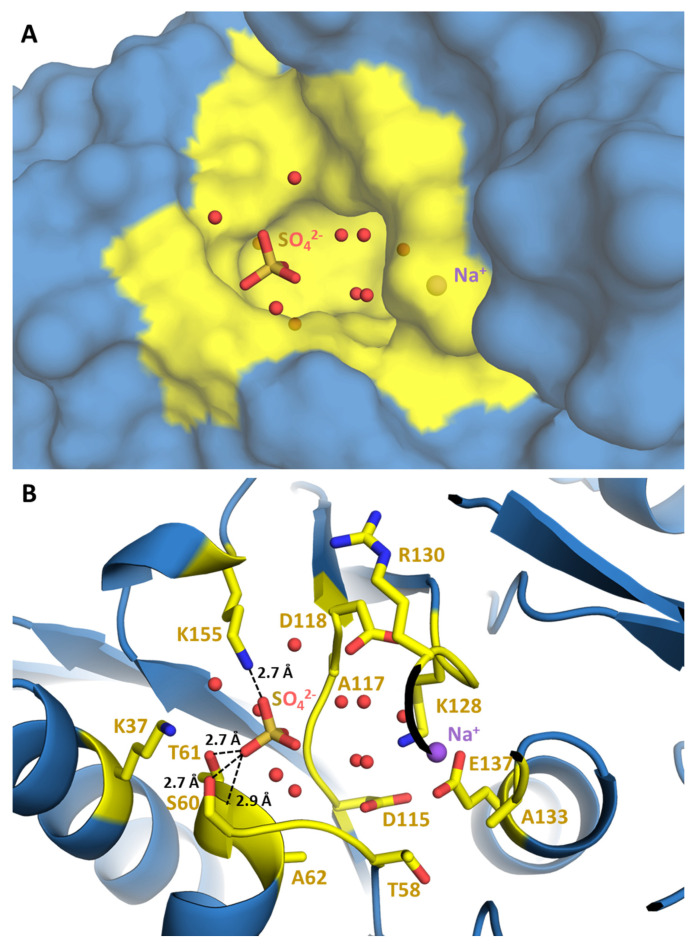
CrRPI1 catalytic pocket. (**A**) The Connolly solvent exclusion surface of the catalytic cleft of CrRPI1 with ligands in it (sulfate ion, water, sodium ion). Residues contributing to the cavity are colored in yellow, other residues are colored in blue. (**B**) The same view of the catalytic cleft as in (**A**). The protein is colored in blue and represented in cartoon. Residues contributing to the catalytic cleft are colored in yellow and their side chains are represented in sticks. Polar contacts between ligand and residues are represented in black dashed lines with corresponding interatomic distances indicated in Ångströms (Å).

**Figure 5:**
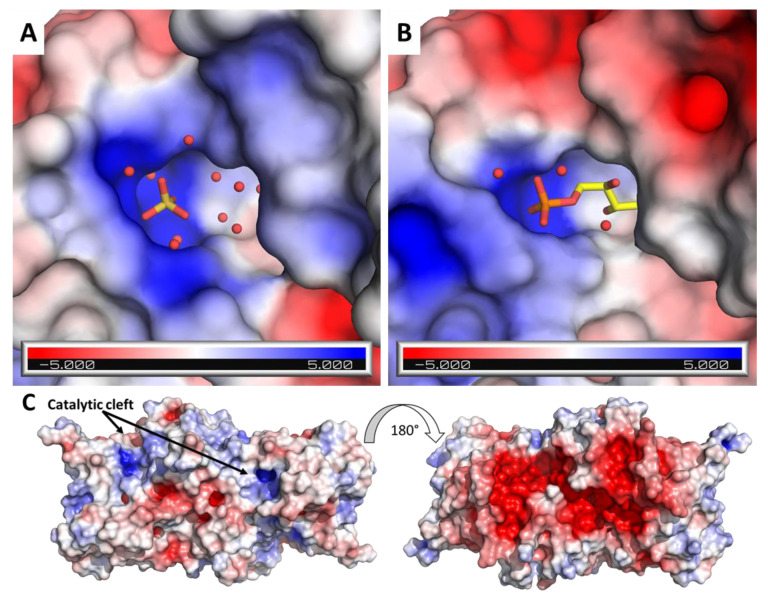
Electrostatic potential of RPI surface. (**A**) View of the catalytic cleft of CrRPI1 electrostatic surface calculated by PyMOL APBS and represented in a gradient from blue (electropositive) to red (electronegative). The sulfate ion is represented in sticks and water molecules as spheres. (**B**) View of the catalytic cleft of *Legionella pneumophila* RpiA of PDB model 6MC0, superposed to CrRPI1 model and colored as in A. Co-crystallized ribose-5-phosphate is represented in sticks. (**C**) Electronegative patch of CrRPI1 dimer. Electrostatic surface of CrRPI1 dimer facing the catalytic cleft up and with a 180° rotation from this position was calculated by PyMOL APBS.

CrRPI1 crystallographic model includes a sulfate ion, a sodium ion and water molecules located in a shallow pocket exposed to solvent (Figure 4A). Structural superposition of CrRPI1 with RpiA from *Legionella pneumophila* (LpRPI, PDB: 6MC0, RMSD = 0.734 Å), which is co-crystalized with ribose-5-phosphate bound to the active site, reveals extended similarities (Figure S2). Surface charges were calculated on Pymol using the APBS electrostatics plugin [27,28]. For both enzymes, the active site is an analog cavity with an electronegative charge at the center, surrounded by electropositive charges at the rims (Figure 5A,B). Electrostatic surface potentials are similarly distributed on both active sites, suggesting an identical electrostatic complementarity toward the substrate. Indeed, an electropositive patch just outside the cavity is ideally positioned to accommodate the phosphate moiety of R5P in LpRPI (Figure 5B) and was found to anchor the co-crystallized sulfate ion in CrRPI1 (Figure 5A). The sulfate ion in CrRPI1 hence occupies the location of the phosphate of the ribose-5-phosphate (Figure 5A,B) supporting a conserved mode of substrate recognition. This sulfate ion is coordinated by polar contacts with the side chains of S60, T61, and K155 and the main chain amine of T61. Residues K37, G59, S60, T61, A62, I150, and K155 principally contribute to the rest of the pocket surface in CrRPI1 (Figure 4B). All these residues are highly conserved in all species from the three kingdoms of life (Figure S1). Water molecules in CrRPI1 are placed where R5P carbon skeleton binds LpRPI. The sodium ion co-crystallized with CrRPI1 lies where the aldehyde function of R5P binds LpRPI. Residues D115 and E137 have been proposed to play a critical role in the catalytic mechanism [3,29]. In our model of CrRPI1, their side chains are positioned at, respectively, 3.1 Å and 6.4 Å away from the sodium ion, allowing their interaction with the carbonyl function of the substrate. Side chains rearrangements and water displacements are expected for the formation of the Michaelis complex and the subsequent catalytic isomerization.

### 2.4. CrRPI1 Sites of Post-Translational Modifications

All CBC enzymes undergo diverse types of redox post-translational modifications including disulfide bond formation, glutathionylation or nitrosylation, under the control of thioredoxins (TRX) and glutaredoxins (GRX) [15,20]. CrRPI1 contains four cysteines: C149, C250 and C192 are conserved in green microalgae, while C175 is present in algae and land plants (Figure S1). Two cysteines (C149 and C175) were identified as reactive in vivo through a high-throughput screen, and C149 was further identified as a putative TRX target [20]. CrRPI1 was also identified as a target of S-nitrosylation or S-glutathionylation in proteomic studies [16,30]. In our CrRPI1 crystal structure, C149 and C250 position their sulfur atoms 4.4 Å apart, a distance compatible with the formation of a disulfide bridge. C149 and C250 side chains are modelled as free thiols (Figure 1A and Figure S3). Both side chains are buried in the protein hydrophobic core where TRX or GRX should be excluded once RPI1 is folded. The two other cysteines of CrRPI1 are closer to the surface but separated by 20 Å, precluding the formation of an intramolecular disulfide bridge. C192 has a solvent exposed backbone and its sidechain points toward the hydrophobic core. C175 has its sulfur atom exposed to solvent and is the most likely to be the target of redox post translational modifications, such as the formation of an intermolecular disulfide bridge with another dimer of CrRPI1 or with a partner protein. Indeed, CrRPI1 is predicted to interact with ten other proteins in *Chlamydomonas reinhardtii* by the STRING database [31] and a disulfide bridge may stabilize or condition these interactions. Phosphoglycerate kinase 1 (PGK1), transketolase 1 (TRK1), ribulose-5-phosphate 3-epimerase 1 (RPE1) and 2 (RPE2), and phosphoribulokinase (PRK) are predicted to belong to this interaction network and are also part of the CBC and proven targets of redox regulation [20,32,33]. Protein interactions may hence contribute to the redox regulation of the CBC under varying physiological and environmental conditions. Furthermore TRK, RPI, RPE, and PRK activities are consecutive in the cycle, which may allow them to constitute an enzymatic metabolon.

A large electronegative patch is present at the back side of the dimer, 20 Å away from the catalytic pocket (Figure 5C). It spans both subunits and forms a continuous ellipsoid of approximately 55 Å in length and 30 Å in width. This extended surface patch is a candidate docking zone for additional partner or regulatory proteins. For instance, photosynthetic redox activator CrTRXf2 was reported to expose an electropositive crown of residues around its active site [34] and a large electropositive crescent surrounds CrPRK active site [35]. Such putative interactants or others bearing similar electropositive surfaces would favorably interact on the back side of CrRPI1 by electrostatic complementarity.

Phosphoproteomic studies [21] identified two phosphorylated amino acids on CrRPI1: T61 and S87 (Figure S3). Both are localized on the same surface as the catalytic cavity, T61 hydroxyl group being at 2.7 Å of one of the oxygens of aforementioned active site sulfate. S87 is positioned 13.2 Å away from T61, at the opposite side of the catalytic cleft at 7.1 Å of E137. Phosphorylation of T61 would insert a bulky negatively charged group, which would probably preclude the binding of phosphate from the R5P substrate. Phosphorylation of T61 would thus outcompete the binding of R5P. S87 phosphorylation may play a less direct role in the regulation of R5P binding. Both T61 and S87 are fully conserved from bacteria to plants and metazoans (Figure S1).

## 3. Discussion

We describe the first structure of a ribose-5-phosphate isomerase from a *Viridiplanta*, the model microalga *Chlamydomonas reinhardtii*. The structural fold of CrRPI1 (Figure 1B) is similar to previously described structures of canonical RPI_A_ [29] even though plastidial RPI participates in both the Calvin-Benson cycle and the pentose phosphate pathway. CrRPI1 catalytic site is described free from substrate and product, representing the apo state of the enzyme. Six water molecules, a sulfate ion and a sodium ion are however present and therefore allow to predict the coordination and placement of its substrate R5P in this pocket through structural superposition and sequence analysis. Most of the residues shaping the catalytic pocket are strictly conserved (Figure S1), supporting a conserved catalytic mechanism for all RPI_A_ described to date [4].

CrRPI1 is a homodimer in solution and in the crystal packing (Figure 1A, Figure 2A,B, and Figure 3A–C). This homodimeric organization was previously observed (*L. pneumophila* PDB 6MC0). RPI was alternatively reported to form homotetramers in other species such as *Saccharomyces cerevisiae* or archaea [36,37]. The oligomerization appears to depend on the length of the 197–204 loop and the interaction network of the conserved residue R197, as suggested based on the *S. cerevisiae* structure [36]. In CrRPI1 homodimer, the distance between catalytic residues D115_A_ and D115_B_ is 40 Å. Whether homodimerization induces cooperativity between the active sites will be tested in future studies.

By contributing to the Calvin-Benson cycle and to the pentose phosphate pathway [2,38], CrRPI1 activity should be subject to several, maybe mutually exclusive, regulations of its catalytic parameters. Structural analysis suggests several possible, mutually permissive regulation mechanisms. First, CrRPI1 was identified in *C. reinhardtii* thioredoxome [20], as a consequence of its affinity with the dithiol-disulfide exchanger thioredoxin. Hence, RPI1 is likely to be activated in concert with other CBC enzymes, such as PRK [35]. The presence of only cysteines 149 and 250 at disulfide bonding distance, but not accessible to solvent, argues in favor of irreversible redox states of the cysteine pair once the protein is folded. The two other cysteines 175 and 192 side chains are close to or exposed to solvent. They are modelled in CrRPI1 at a distance of 20 Å which precludes the formation of an intramolecular disulfide bridge. Alternatively, other redox post translational modifications of these cysteines, such as intermolecular disulfide bridges, S-glutathionylation, S-nitrosylation may occur as suggested by proteomic studies [16,20,30]. Interestingly, RPI1 could also interact with partner proteins, including through intermolecular disulfide bridges. The formation of multienzymatic complexes with following and preceding enzymes from the CBC may allow channeling of substrates as suggested by a study on flux distribution and metabolic pools in *Chlamydomonas reinhardtii* chloroplast [39]. This hypothesis has to be tested by isolation of such complexes from cellular extracts.

Regulation of the isomerase activity could also be achieved by phosphorylation of residues T61 and S87. Addition of a phosphate group onto T61 hydroxyl would block the entrance of the catalytic pocket with a negatively charged steric hindrance at the R5P and Ru5P binding site. This hypothesis seems attractive as, most importantly, these two residues are highly conserved throughout all analyzed species. This might indicate a ubiquitous, ancestral regulation rather than a CBC-specific control. The identity of the kinase/phosphatase couple that specifically targets CrRPI1 will be addressed during future research.

In spite of its long evolution in the photosynthetic lineage, chloroplastic CrRPI1 seems to have no major structural difference with previously described RPI of the A-type. Some putative regulatory elements are diverging from other studied RPI_A_ and will need further functional characterizations. The co-regulations exerted by multiples signaling pathways on RPI, the coordinated regulations on RPI, and the rest of the metabolic pathways it belongs to should be further computed in a systems biology perspective. For this purpose, our high-resolution crystal structure of the first chloroplastic ribose-phosphate isomerase serves as a general blueprint for the analytic and synthetic biochemistry of an essential step of photosynthesis.

## 4. Materials and Methods

### 4.1. Cloning

The amino acid sequence of *Chlamydomonas reinhardtii* ribose-5-phosphate isomerase (Cre03.g187450, UniRef100 entry A8IRQ1) was analyzed with TargetP2.0 [40], ChloroP [41] and Predalgo [8] to predict the transit peptide cleavage site. The subsequent mature sequence of chloroplastic protein coding for amino acids 23 to 269 was amplified by PCR using a forward primer introducing an NcoI restriction site (bolded) at the start codon: 5′-CGCA**CCATGG**CCGCGCCGGTCTCAA-3′ and a reverse primer introducing a BamHI restriction site (bolded) downstream of the stop codon: 5′-GCACG**GGATCC**TTAGTGCTTCTTGGGGTTGGG-3′ as previously reported [30]. The PCR was performed using the *Chlamydomonas reinhardtii* EST index [42] database plasmid AV390085 obtained from the Kazusa DNA Research Institute (Chiba, Japan). After purification, the PCR product was digested by NcoI and BamHI and cloned into the NdeI and BamHI restriction sites of a modified pET-3d-His vector allowing expression of the protein with a polyhistidine-tag at the N-terminus. The resulting plasmid, pET3d-His-CrRPI1 was then validated by Sanger sequencing.

### 4.2. Protein Expression and Purification

Recombinant CrRPI1 was produced using the *Escherichia coli* strain BL21(DE3) Rosetta-2 pLysS (Novagen Merck, Darmstadt, Germany) transformed with the pET3d-His6-CrRPI1 plasmid. Bacteria were grown in LB medium supplemented with ampicillin (100 µg/mL) at 37 °C until the culture reached exponential phase at an optical density at 600 nm of 0.4. The expression of RPI was then induced by addition of 0.2 mmol/L of IPTG and incubation at 27 °C for three hours. Cells were harvested by centrifugation 5000 rcf for 10 min at 4 °C, resuspended in 30 mmol/L Tris-HCL pH = 7.9, 0.5 mmol/L EDTA (buffer A), and lysed by 2 min sonication with 0.4 sec pulses at output 5 on W-375 sonicator equipped with microtip (Qsonica, Newtown, CT, USA). The total extract was then centrifuged at 20,000 rcf for 20 min and the soluble fraction loaded on an affinity chromatography with 2 mL of NiNTA resin (Sigma-Aldrich Merck, Darmstad, Germany). The resin was washed with Buffer A supplemented with increasing concentrations of imidazole (10 mmol/L, 20 mmol/L, and 30 mmol/L). CrRPI1 was then eluted in with buffer A supplemented with 100 mmol/L, 200 mmol/L and 300 mmol/L of imidazole. Fractions were then analyzed by SDS-PAGE on a 12% acrylamide gel revealed by Coomassie blue staining. Fractions containing purified CrRPI1 were pooled, and buffer exchanged against buffer A prior to being concentrated by ultrafiltration on 3000 MWCO filter units (Millipore Merck, Darmstadt, Germany). A final concentration of 9 mg.mL^−1^ was measured by NanoDrop 2000 spectrophotometer (Thermo Fisher Scientific, Waltham, MA, USA) with theoretical Mw = 27,551 g.mol^−1^ and ε = 14,565 mol^−1^.L.cm^−1^. Proteins were then analyzed by injection on a Superose 6 increase size exclusion chromatography column in buffer C (20 mM Tris-HCl pH 7.9, 100 mmol/L NaCl).

### 4.3. Protein Crystallization and Structure Determination

Purified CrRPI1 was tested for crystallization on commercial sparse-screening conditions (Qiagen, Hilden Germany) of the Joint Center for Structural Genomics screens [43] and JBScreen classic 1–8 (Jena Bioscience, Jena, Germany) with a mixture of 50 nL of protein and 50 nL of precipitant solution equilibrated against 30 µL of reservoir solution at 20 °C. Two monocrystal grew after one year in conditions JBScreen 6-C8 (3 mmol.L^−1^ ammonium sulfate) and JBScreen 6-D9 (3 mmol.L^−1^ ammonium sulfate, 20% glycerol). Crystals were flash frozen in liquid nitrogen for diffraction experiments at Proxima-1 beamline of SOLEIL synchrotron (Gif sur Yvette, France) [44]. A 99.63% complete dataset at 1.40 Å resolution was obtained from 3600 images over 360° of phi rotation and indexed in the P2_1_ space group, integrated, scaled and converted with XDSME [45]. Structure was phased by molecular replacement with PHENIX [46,47], PHASER-MR [48] using a search model obtained from the Robetta server [49]. Two CrRPI1 monomers were found in the asymmetric unit. Model was then refined by iterative cycle of manual building in WinCOOT [50,51], followed by refinement with PHENIX.REFINE [52] until completion of a structure passing MOLPROBITY [53] evaluation with 100% residues in Ramachandran restrains, RMS(bond) = 0.011, RMS(angles) = 1.18 and final Rwork = 0.1594, Rfree = 0.1774 (Table 1). Structure representations were drawn with PYMOL (Schrodinger, New York, NY, USA).

### 4.4. SEC-SAXS Analysis

Here, 50 µL of pure CrRPI1 at a concentration of 10 mg.mL^−1^ were injected on BioSEC-3 300 size-exclusion chromatography column (Agilent Technologies, Santa Clara, CA, USA) equilibrated in buffer C, in series with the small-angle X-rays scattering (SAXS) exposure capillary at the synchrotron beamline SWING (SOLEIL, Gif sur Yvette, France). Collected scattering images were analyzed on the application Foxtrot 3.3.4 (Xenocs, Sassenage, France) and the ATSAS 2.8.3 suite [54,55]. PrimusQT calculated a radius of gyration of 28.16 ± 0.18 Å and estimated SAXS CrRPI1 molecular weight at 52,781 Da (Qp), 53,991 Da (MoW), 49,221 (Vc), and 55,257 Da (size and shape). This molecular weight allows us to fit between 1.78 and 2.00 times the molecular weight of monomeric recombinant CrRPI1 estimated with Expasy ProtParam at 27,551 Da.

### 4.5. Structural Data

Crystallographic data are registered at the Protein Data Bank under accession code 6ZXT.

## Figures and Tables

**Table 1:** Crystallographic data collection and refinement statistics.

	CrRPI1
Wavelength (Å)	0.979
Resolution range (Å)	40.22–1.40 (1.45–1.40)
Space group	P 2_1_
Unit cell	45.15 63.95 80.91 90 96.13 90
Total reflections	613,155 (55,349)
Unique reflections	89,012 (8611)
Multiplicity	6.9 (6.4)
Completeness (%)	99.6 (96.5)
Mean I/sigma(I)	20.58 (1.24)
Wilson B-factor (Å^2^)	21.45
R-merge	0.0519 (1.272)
R-meas	0.05611 (1.382)
R-pim	0.02114 (0.5327)
CC1/2	0.999 (0.382)
CC*	1 (0.744)
Reflections used in refinement	88,983 (8601)
Reflections used for R-free	2000 (194)
R-work	0.1594 (0.3457)
R-free	0.1774 (0.3813)
CC (work)	0.972 (0.730)
CC (free)	0.967 (0.836)
Number of non-hydrogen atoms	4206
macromolecules	3648
ligands	47
solvent	511
Protein residues	474
RMS (bonds) (Å)	0.011
RMS (angles) (deg)	1.18
Ramachandran favored (%)	99.79
Ramachandran allowed (%)	0.21
Ramachandran outliers (%)	0.00
Rotamer outliers (%)	1.00
Clashscore	2.69
Average B-factor (Å^2^)	25.46
macromolecules	23.78
ligands	47.04
Solvent	35.49

Statistics for the highest-resolution shell are shown in parentheses.

## Supplementary Materials

Supplementary materials can be found at https://www.mdpi.com/1422-0067/21/20/7787/s1. Figure S1. Sequence alignment made by Clustal Omega of RPI_A_ from different species. Residues with more than 50 % conservation are colored in red, strictly conserved residues are in white with a red background. Numbering is according to *Chlamydomonas reinhardtii.* RPI sequences were retrieved from uniprotKB database for the following species: *Chlamydomonas reinhardtii* (A8IRQ1), *Glycine Max* (I1NC83)*, Arabidopsis thaliana* (Q9S726)*, Plasmodium falciparum* (Q8I3W2), *Deinococcus radiodurans* (Q9RW24)*, Streptococcus pneumoniae* (B2INU5)*, Mus musculus* (P47968), *Homo sapiens* (P49247)*, Gonium pectorale* (A0A150H4N9)*, Volvox carteri f. nagariensis* (D8TV46)*, Chlorella sorokiniana* (A0A2P6TRF5)*, Raphidocelis subcapitata* (A0A2V0PB57)*, Chlorella variabilis* (E1Z7C4)*, Chlamydomonas eustigma* (A0A250XAH6)*, Polytomella sp. Pringsheim 198.80* (K0J8U0)*, Thermus thermophilus* (Q72J47)*, Synechocystis sp. PCC6803* (Q55766)*, Burkholderia thailandensis* (A0A2Z4SMZ7)*, Legionella pneumophila* (A5I9R2). Figure S2. Comparison of the catalytic clefts of CrRPI1 and LpRPI (PDB entry 6MC0). (A) Structures are aligned (RMSD= 0.701 Å) and represented in cartoon with side chains of catalytic pocket colored in magenta and represented as sticks. Other residues of CrRPI1 and LpRPI are respectively colored in blue and green. Sulfate ion and Ribose-5-phosphate are represented in sticks, water molecules and Na ion are represented in spheres. (B) Structure of CrRPI1. (C) Structure of LpRPI. Figure S3. (A) CrRPI1 post-translational modifications sites. Residues of the catalytic cleft are colored in orange. Cysteines and residues found phosphorylated in Wang, Gau et al. are represented in sticks and respectively colored in red and blue. (B) Connolly solvent exclusion surface of the same view as A. and same color code.
