# Supplementary material for: High-Resolution Crystal Structure of Chloroplastic Ribose-5-Phosphate Isomerase from Chlamydomonas reinhardtii—An Enzyme Involved in the Photosynthetic Calvin-Benson Cycle
Source: Int J Mol Sci. 2020 Oct 21;21(20):7787. doi: 10.3390/ijms21207787 (PMC7589169; doi:10.3390/ijms21207787)
Supplement: Supplementary file 1 [file ijms-21-07787-s001.pdf]

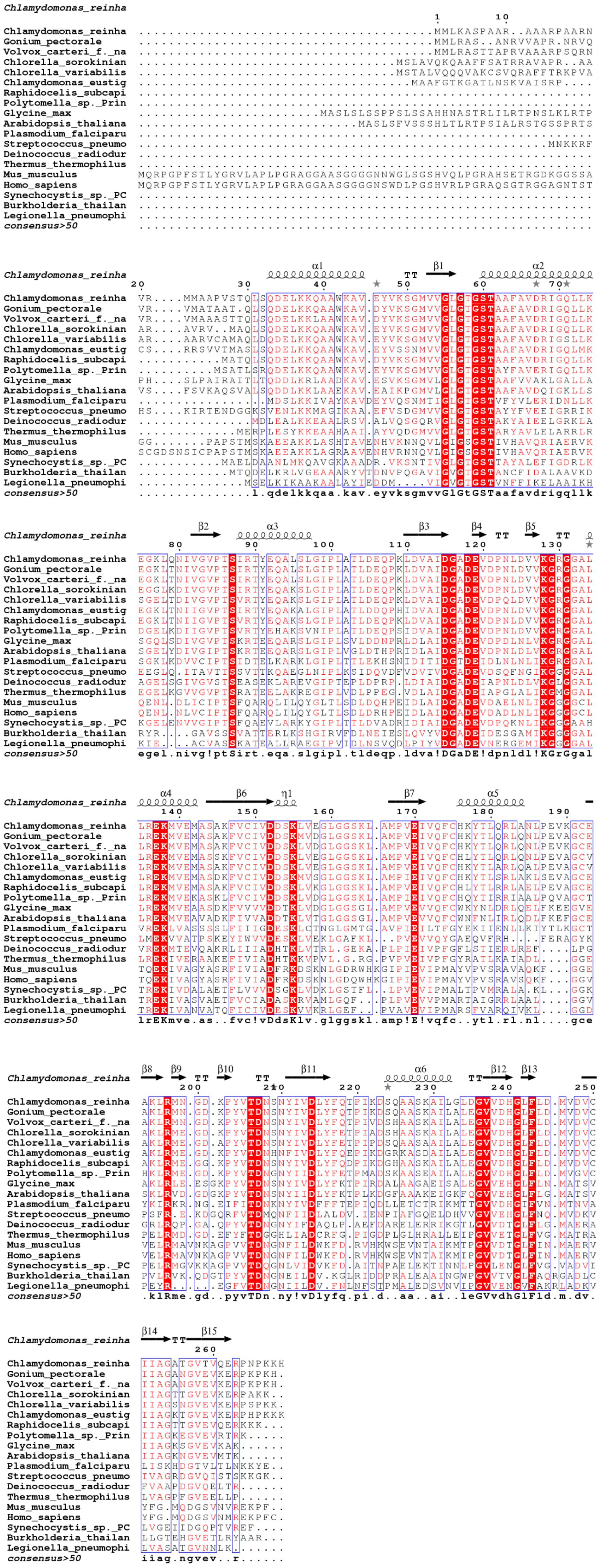

<u>Supplementary figure S1:</u> Sequence alignment made by Clustal Omega of RPI_A from different species. Residues with more than 50 % conservation are colored in red, strictly conserved residues are in white with a red background. Numbering is according to *Chlamydomonas reinhardtii*. RPI sequences were retrieved from uniprotKB database for the following species : *Chlamydomonas reinhardtii* (A8IRQ1), *Glycine Max* (I1NC83), *Arabidopsis thaliana* (Q9S726), *Plasmodium falciparum* (Q8I3W2), *Deinococcus radiodurans* (Q9RW24), *Streptococcus pneumoniae* (B2INU5), *Mus musculus* (P47968), *Homo sapiens* (P49247), *Gonium pectorale* (A0A150H4N9), *Volvox carteri f. nagariensis* (D8TV46), *Chlorella sorokiniana* (A0A2P6TRF5), *Raphidocelis subcapitata* (A0A2V0PB57), *Chlorella variabilis* (E1Z7C4), *Chlamydomonas eustigma* (A0A250XAH6), *Polytomella sp. Pringsheim 198.80* (K0J8U0), *Thermus thermophilus* (Q72J47), *Synechocystis sp. PCC6803* (Q55766), *Burkholderia thailandensis* (A0A2Z4SMZ7), *Legionella pneumophila* (A5I9R2).

A

B

C

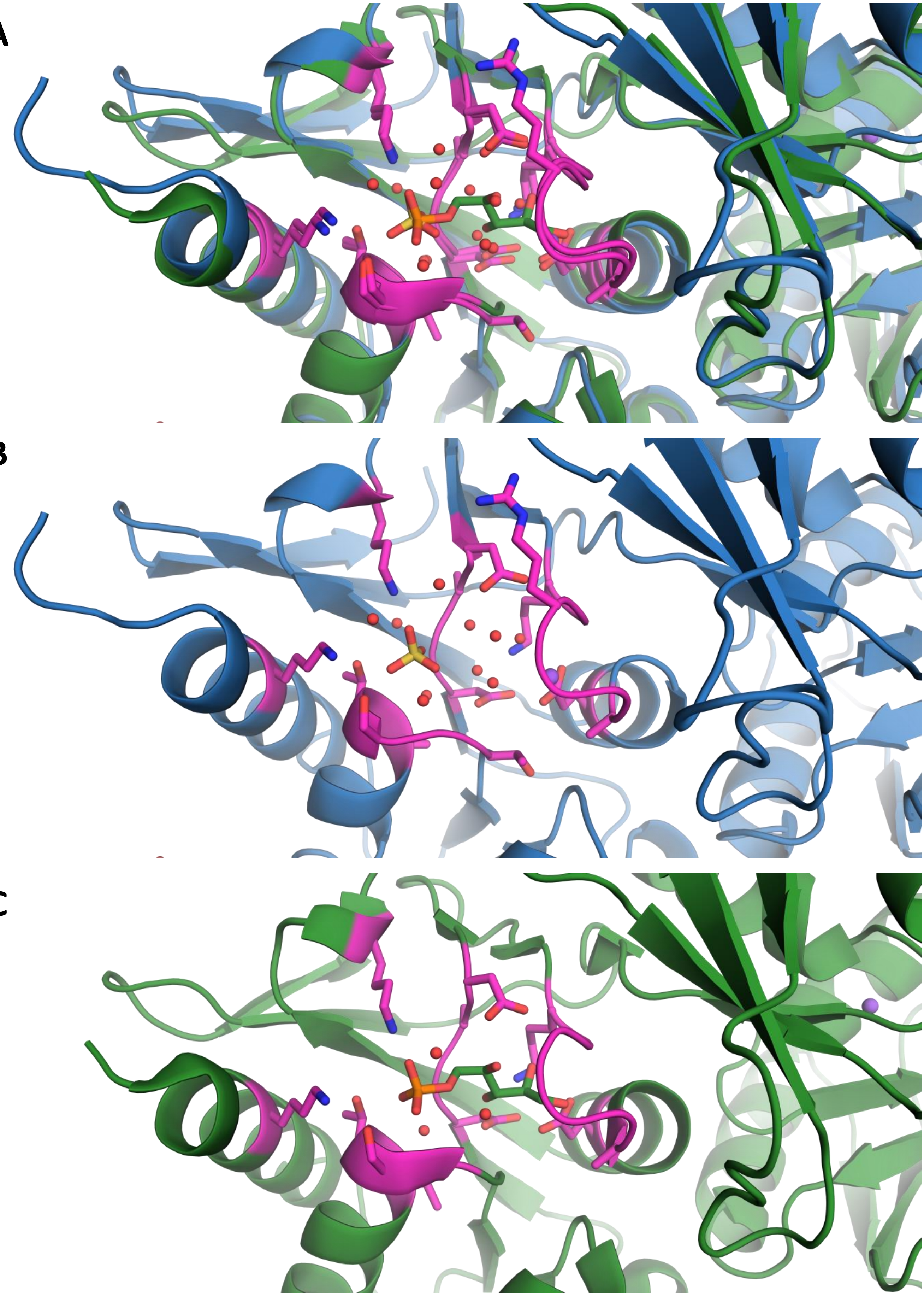

<u>Supplementary figure S2:</u> Comparison of the catalytic clefts of CrRPI1 and LpRPI (PDB entry 6MC0). (**A**) Structures are aligned (RMSD= 0.701 Å) and represented in cartoon with side chains of catalytic pocket colored in magenta and represented as sticks. Other residues of CrRPI1 and LpRPI are respectively colored in blue and green. Sulfate ion and Ribose-5-phosphate are represented in sticks, water molecules and Na ion are represented in spheres. (**B**) Structure of CrRPI1. (**C**) Structure of LpRPI.

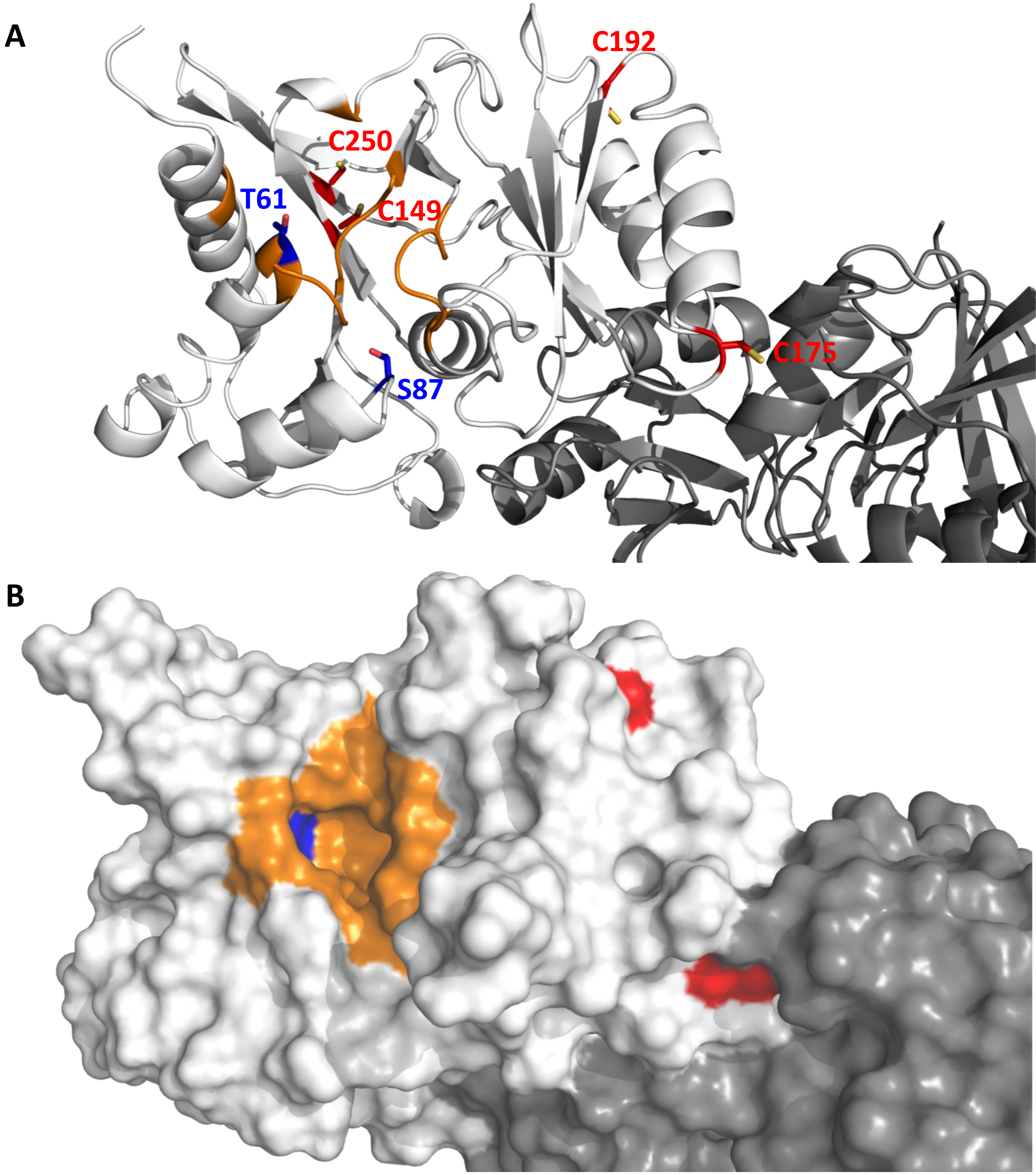

<u>Supplementary figure S3:</u> (**A**) CrRPI1 post-translational modifications sites. Residues of the catalytic cleft are colored in orange. Cysteines and residues found phosphorylated in Wang, Gau *et al.* are represented in sticks and respectively colored in red and blue. (**B**) Connolly solvent exclusion surface of the same view as A. and same color code.
